# Evaluation of malaria outbreak detection methods, Uganda, 2022

**DOI:** 10.1186/s12936-024-04838-w

**Published:** 2024-01-13

**Authors:** Marie Gorreti Zalwango, Jane F. Zalwango, Daniel Kadobera, Lilian Bulage, Carol Nanziri, Richard Migisha, Bosco B. Agaba, Benon Kwesiga, Jimmy Opigo, Alex Riolexus Ario, Julie R. Harris

**Affiliations:** 1 Uganda Public Health Fellowship Programme-Uganda National Institute of Public Health, Kampala, Uganda; 2 National Malaria Control Division, Ministry of Health, Kampala, Uganda; 3 Division of Global Health Protection, US Centers for Disease Control and Prevention, Kampala, Uganda

**Keywords:** Malaria, Malaria outbreak, Epidemic thresholds, Uganda

## Abstract

**Background:**

Malaria outbreaks are detected by applying the World Health Organization (WHO)-recommended thresholds (the less sensitive 75th percentile or mean + 2 standard deviations [2SD] for medium-to high-transmission areas, and the more sensitive cumulative sum [C-SUM] method for low and very low-transmission areas). During 2022, > 50% of districts in Uganda were in an epidemic mode according to the 75th percentile method used, resulting in a need to restrict national response to districts with the highest rates of complicated malaria. The three threshold approaches were evaluated to compare their outbreak-signaling outputs and help identify prioritization approaches and method appropriateness across Uganda.

**Methods:**

The three methods were applied as well as adjusted approaches (85th percentile and C-SUM + 2SD) for all weeks in 2022 for 16 districts with good reporting rates ( ≥ 80%). Districts were selected from regions originally categorized as very low, low, medium, and high transmission; district thresholds were calculated based on 2017–2021 data and re-categorized them for this analysis.

**Results:**

Using district-level data to categorize transmission levels resulted in re-categorization of 8/16 districts from their original transmission level categories. In all districts, more outbreak weeks were detected by the 75th percentile than the mean + 2SD method (p < 0.001). For all 9 very low or low-transmission districts, the number of outbreak weeks detected by C-SUM were similar to those detected by the 75th percentile. On adjustment of the 75th percentile method to the 85th percentile, there was no significant difference in the number of outbreak weeks detected for medium and low transmission districts. The number of outbreak weeks detected by C-SUM + 2SD was similar to those detected by the mean + 2SD method for all districts across all transmission intensities.

**Conclusion:**

District data may be more appropriate than regional data to categorize malaria transmission and choose epidemic threshold approaches. The 75th percentile method, meant for medium- to high-transmission areas, was as sensitive as C-SUM for low- and very low-transmission areas. For medium and high-transmission areas, more outbreak weeks were detected with the 75th percentile than the mean + 2SD method. Using the 75th percentile method for outbreak detection in all areas and the mean + 2SD for prioritization of medium- and high-transmission areas in response may be helpful.

## Background

The global technical strategy for malaria 2016–2030 of the World Health Organization (WHO) recommends strengthening malaria surveillance as a fundamental activity to inform programme planning and implementation for improved outbreak detection in malaria-endemic countries [1]. According to the World Malaria Report of 2022, Uganda is ranked as the third-highest contributor to malaria burden globally, with 95% of the country being highly endemic and 5% prone to malaria epidemics [2, 3].

A malaria outbreak is characterized as an increase in case counts above the threshold for the normal seasonal pattern of malaria in an area. This threshold is usually calculated based on historical routine data at the district level for a minimum of 5 years [4, 5]. The WHO recommends various methods to calculate thresholds, including the 75th percentile, mean ± 2 standard deviations (SD), cumulative sum (C-SUM), and constant case counts [4]. The 75th percentile method considers the threshold as the 75th percentile of the average number of cases for a specific epidemiological week in that district over the past 5 years. The mean + 2SD method takes the mean number of cases for that week over the last 5 years and adds 2SD to establish the threshold. The C-SUM method involves a running average of cases for the current epi week, the previous week, and the following week over the past 5 years [4]. To accommodate seasonal malaria peaks that are not necessarily epidemics, modifications to these methods have been proposed, including raising the 75th percentile to the 85th percentile, and increasing the C-SUM method threshold by adding two standard deviations (C-SUM + 2SD) [4]. These adaptations are meant to improve the ability to distinguish between true outbreaks and regular seasonal variations.

The threshold calculation method that is recommended depends on the extent of malaria transmission in a given area. The WHO defines high transmission as an annual parasite index (API) > 450/1000, medium transmission as 251–450/1000 API, low transmission as 101–250/1000 API, and very low transmission as ≤ 100/1000 API [4]. The C-SUM method is recommended for areas with very low to low transmission; however, it is considered too sensitive for outbreak detection in medium- to high-transmission areas [4]. In the medium- to high-transmission areas, the 75th percentile method and mean + 2SD methods are both recommended by the WHO; however, they are considered too insensitive to accurately detect outbreaks in low-transmission areas [4]. For any method used, a malaria epidemic is declared when the malaria cases are above the threshold for > 2 weeks consecutively. Uganda’s malaria epidemic preparedness and response plan for 2019 suggests using the 75th percentile method at the national level and for all districts [6]. However, some districts use the mean + 2SD and others use the 75th percentile methods, based on the WHO recommendation for similar settings.

From 2019 to 2022, Uganda’s health information system reported a rise in confirmed malaria cases [7]. During the first half of 2022, more than half of the districts in Uganda were in outbreak mode for at least 10 weeks, according to the 75th percentile method used [8]. While every outbreak should be investigated and responded to by the national rapid response team, limited resources for logistics and human resources forced the national malaria control programme to restrict its response to only a few districts, using the number of complicated malaria presentations and malaria deaths as the prioritization measure. With the rate of progress slowing in terms of malaria control, not only in Uganda but also in other sub-Saharan African countries [9, 10], there will be a need to ensure that appropriate methods are being used to identify malaria outbreaks and that prioritization methods are available when sufficient resources are not. The three threshold approaches were evaluated to compare their outbreak-signaling outputs in Uganda for improved malaria epidemic detection and response.

## Methods

### Study setting

Uganda comprises 15 health regions, of which 2 (West Nile and Acholi Regions) are considered areas with high annual malaria transmission rates. Five (Lango, Karamoja, Teso, Bukedi, and Busoga Regions) are considered medium malaria transmission areas and seven (South Central, North Central, Kampala, Ankole, Tooro, Bugisu and Bunyoro Regions) are considered low malaria transmission areas. Kigezi Region is considered to have very low malaria transmission and is targeted for malaria elimination in the Uganda National Malaria Strategic Plan 2025 [4, 7, 11].

## Data source

Historic weekly malaria surveillance data from the District Health Information System version 2 (DHIS2) during 2017–2021 was used for the calculation of thresholds. The health facility malaria data are routinely generated at health facilities in outpatient registers. The data are aggregated weekly into health facility weekly surveillance reports, which are submitted to the DHIS2 using a short message system (SMS). This captures information for all health facilities in the districts. The weekly reporting rates for the districts can also be calculated based on data from this system using submitted reports (numerator) divided by expected reports (denominator). Districts with reporting rates of < 80% are considered to have incomplete data submitted.

## Study variables, data abstraction, and analysis

Pivot tables were used to filter secondary data on weekly confirmed malaria cases by both rapid diagnostic test (RDT) and microscopy from the health information management system weekly disease surveillance reports (HMIS 033b report) from 2017 to 2022 available in the DHIS2. Additionally, data on weekly reporting rates for all districts was extracted. Data were extracted for each year for each district. The Ministry of Health (MoH) considers a reporting rate of ≥ 80% as the minimal level for usable data. Sixteen out of 146 districts were selected for the evaluation based on having reporting rates ≥ 80% over the 5-year period and based on their stated regional malaria transmission intensity (four each in the high, medium, low, and very low transmission regions). District API was calculated using malaria cases (numerator) and the total population (denominator) obtained from Uganda Bureau of Statistics census data for the selected districts. Malaria transmission levels by district were re-calculated using district data to enable us evaluate the accuracy of regional-level assignment of transmission levels and evaluate the different threshold approaches accurately.

Using 2022 as the year of review, thresholds were calculated using historic data from 2017 to 2021 for the selected districts. Thresholds were calculated using the three recommended approaches: Mean + 2SD, 75th percentile, and C-SUM to establish their outbreak detection sensitivity, using the highly sensitive C-SUM method as the reference. Case counts were not considered since Uganda is highly endemic for malaria and they are not recommended for such settings [4]. Malaria cases for 2022 were plotted together with the thresholds and displayed using line graphs.

The 85th percentile and C-SUM + 2SD adjusted approaches were also evaluated to see how outbreak week detection changed from the original approaches. The difference in malaria outbreak weeks detected by the various methods were compared for significance using chi-square in STATA software version 14. Finally, the number of outbreak weeks detected by the method used during 2022 and the recommended threshold method were compared, based on the district transmission level. The level of significance was considered at p < 0.05. For graphical presentation in this report, one district was picked randomly from each transmission level category (Fig. 1).

**Fig. 1:**
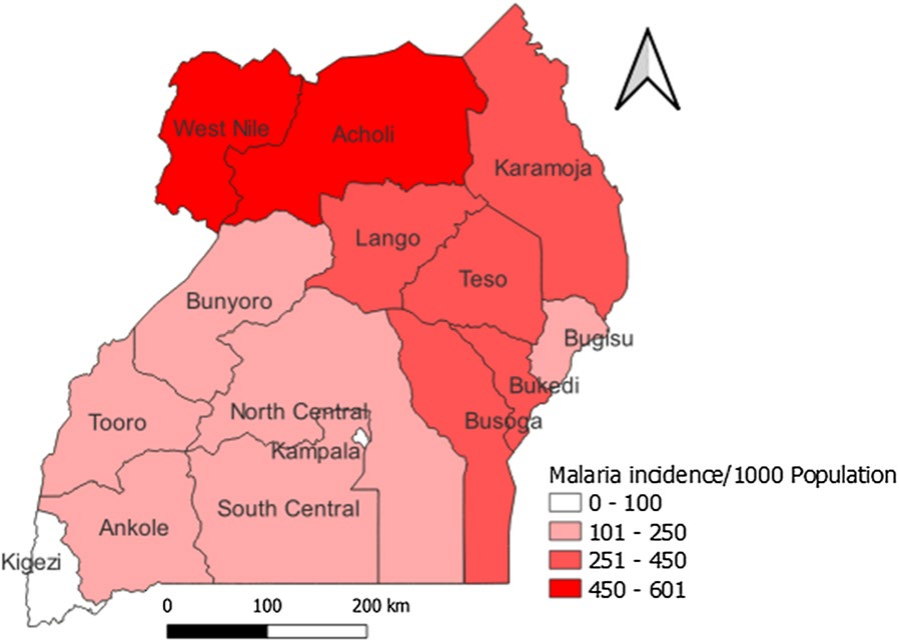
Average regional malaria transmission rates, Uganda, 2017–2021

## Results

### Characteristics of the study data

Varying malaria incidence levels were identified for districts in the same malaria transmission region (Table 1). Overall, 8 of the 16 districts were recategorized based on the use of district data rather than regional data. These included one district (Nwoya) reassigned from ‘high’ to ‘medium’, two districts (Butambala and Bundibugyo) re-categorized from ‘low’ to ‘medium’, 1 district (Kanungu) recategorized from ‘very low’ to ‘low’, two districts (Alebtong and Kibuku) recategorized from ‘medium’ to ‘low’, two districts (Ntoroko and Bukwo) re-categorized from ‘’low’ to ‘very low’. Due to this identified granularity in actual transmission levels, districts were re-categorized by transmission level using district-level data and these assignments were used in the rest of the analysis (Table 1).

**Table 1:** Malaria transmission levels and reporting rates over time for the study districts

Region	District	Original transmission category	Malaria incidence/1000 population					% Reporting rates				
			2017	2018	2019	2020	2021	2017	2018	2019	2020	2021
			High Transmission Regions									
West Nile	Yumbe	High	483	551	659	696	565	89	98	100	97	100
West Nile	Nebbi	High	390	420	741	517	463	81	95	94	87	80
Acholi	Lamwo	High	960	356	567	756	753	81	85	93	92	86
			Medium Transmission Regions									
Acholi	Nwoya	High	264	347	659	473	277	86	84	82	94	100
Karamoja	Moroto	Medium	294	224	389	473	458	80	96	89	90	90
South Central	Butambala	Low	593	309	508	383	241	86	80	95	84	88
Tooro	Bundibugyo	Low	437	300	359	340	380	89	96	95	91	96
			Low Transmission Regions									
Kigezi	Kanungu	Very low	247	148	211	247	219	80	80	80	80	84
Lango	Alebtong	Medium	117	40	72	152	394	80	83	90	87	90
Bukedi	Kibuku	Medium	60	19	25	60	470	89	97	100	96	100
South Central	Mpigi	Low	332	114	168	131	84	97	93	85	80	82
			Very Low Transmission Regions									
Tooro	Ntoroko	Low	100	35	53	88	121	91	91	82	98	89
Kigezi	Rukiga	Very low	42	16	16	14	51	80	83	81	93	100
Kigezi	Kisoro	Very low	31	29	99	32	16	80	84	84	80	84
Kigezi	Rubanda	Very low	24	13	15	16	14	95	97	93	92	97
Bugisu	Bukwo	Low	68	37	70	36	90	92	94	100	89	90

## Outbreak weeks detected per threshold approach and the difference in weeks detected for specific threshold approaches

The number ‘outbreak weeks’ varied by method used across the different transmission levels. For all transmission levels, the difference in malaria outbreak weeks detected by the 75th percentile method and the mean + 2SD was statistically significant, with the 75th percentile method detecting ~ 1.5 to 30 times the number of outbreak weeks as the mean + 2SD method (p < 0.001). In low- and very low-transmission areas, the more sensitive C-SUM method usually detected similar numbers of malaria outbreak weeks as the 75th percentile method. As transmission levels increased, there was a tendency for greater differences between the C-SUM method and the 75th percentile method, with the C-SUM method detecting more outbreak weeks (Table 2). On adjustment of the 75th percentile method to the 85th percentile, there was no difference in the number of outbreak weeks detected for low and medium transmission levels. The adjustment of C-SUM to C-SUM + 2SD reduced its sensitivity to make it equivalent to the mean + 2SD method (Table 2).﻿﻿

**Table 2:** Outbreak weeks detected per threshold approach and the difference in weeks detected for specific threshold approaches for selected districts in Uganda, 2022

Region	District	Total number of outbreak weeks detected per method					Statistical difference in weeks detected by the methods (p-value)			
		C-SUM	75%	Mean + 2SD	C-SUM + 2SD	85%	C-SUM vs. 75th Perc	75% vs. Mean + 2 SD	75% vs. 85%	Mean + 2SD vs. C-SUM + 2SD
		High transmission regions								
West Nile	Yumbe	42	31	2	2	21	0.02	< 0.001	0.04	1
West Nile	Nebbi	32	23	2	2	13	0.08	< 0.001	0.04	1
Acholi	Lamwo	46	33	6	6	26	0.003	< 0.001	0.02	1
		Medium transmission regions								
Acholi	Nwoya	42	30	1	1	17	0.02	< 0.001	0.01	1
Karamoja	Moroto	48	37	10	11	29	0.01	< 0.001	0.1	1
South Central	Butambala	38	20	5	5	14	< 0.001	< 0.001	0.21	1
Tooro	Bundibugyo	45	39	26	25	35	0.14	0.01	0.39	0.84
		Low transmission regions								
Kigezi	Kanungu	35	27	11	11	17	0.06	0.03	0.42	1
Lango	Alebtong	52	50	39	40	49	0.15	0.004	1	0.8
Bukedi	Kibuku	52	52	47	47	52	1	0.05	1	1
South Central	Mpigi	19	21	3	2	9	0.69	< 0.01	1	1
		Very low transmission regions								
Tooro	Ntoroko	50	46	38	37	44	0.37	0.04	0.57	0.83
Kigezi	Rukiga	47	47	29	29	38	1	< 0.001	0.02	1
Kigezi	Kisoro	26	34	0	0	7	0.17	< 0.001	< 0.001	1
Kigezi	Rubanda	20	14	0	0	7	0.14	< 0.001	0.05	1
Bugisu	Bukwo	38	34	12	9	25	0.07	< 0.001	0.08	0.46

75%: 75th percentile; 85%: 85th percentile

## Graphical presentation of malaria outbreak detection in a high-transmission district

**Fig. 2:**
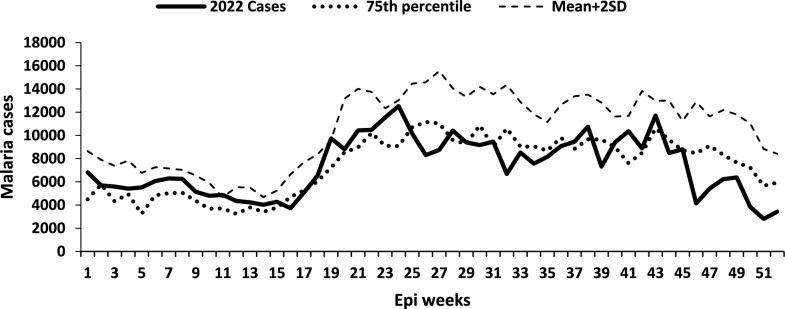
Weekly malaria cases and thresholds on the currently used 75th percentile and mean + 2SD for the year 2022 for the high transmission Yumbe District in West Nile Region, Northern Uganda

The 75th percentile and mean + 2SD methods are both meant to be used for medium- to high-transmission districts. Using Yumbe District (high-transmission district) data, malaria cases using the 75th percentile method exceeded the threshold in 31 weeks compared to 2 (non-sequential) weeks detected by the mean + 2SD method (p-value < 0.001). Since a malaria outbreak is declared with 2 or more sequential outbreak weeks, with mean + 2SD, no malaria outbreak would be detected for Yumbe District. The 75th percentile method classified epidemics from weeks 1–15 and weeks 21–24 (Fig. 2).

## Graphical presentation of malaria outbreak detection in a medium transmission district

**Fig. 3:**
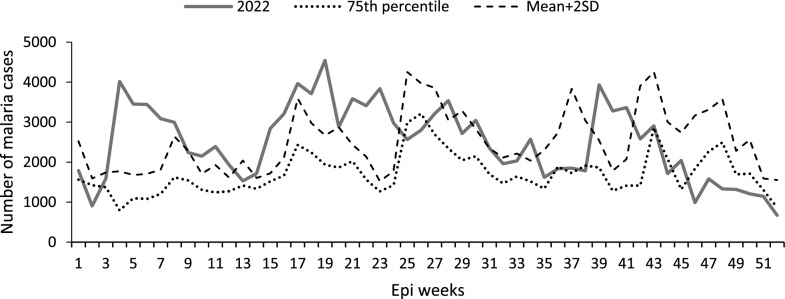
Weekly malaria cases on the currently used 75th percentile and mean + 2SD for the year 2022 for the medium-transmission Bundibugyo District in Tooro Region, Western Uganda

Bundibugyo District, a medium-transmission district, showed 36 weeks exceeding the threshold using the 75th percentile method and 26 weeks using the mean + 2SD method. This would have resulted in the district having a malaria outbreak requiring epidemiologic investigation from weeks 5 to 25 using the mean + 2SD method, and weeks 4–25, 29–36, and 41–43 using the 75th percentile method (Fig. 3).

## Graphical presentation of malaria outbreak detection in a low malaria transmission district

**Fig. 4:**
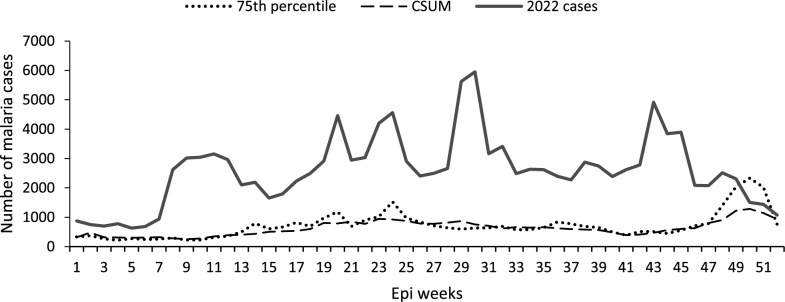
Weekly malaria cases on the currently used 75th percentile and C-SUM for the year 2022 for the low transmission Alebtong District in Lango Region, Northern Uganda

Alebtong District, a low-transmission district, showed 50 weeks exceeding the threshold using the 75th percentile method and 52 weeks using the C-SUM method. The district would have had a malaria outbreak requiring epidemic investigation for 49 weeks in 2022 using the 75th percentile method, and 52 epidemic weeks using the C-SUM method (Fig. 4).

## Graphical presentation of malaria outbreak detection in a very-low malaria transmission district

**Fig. 5:**
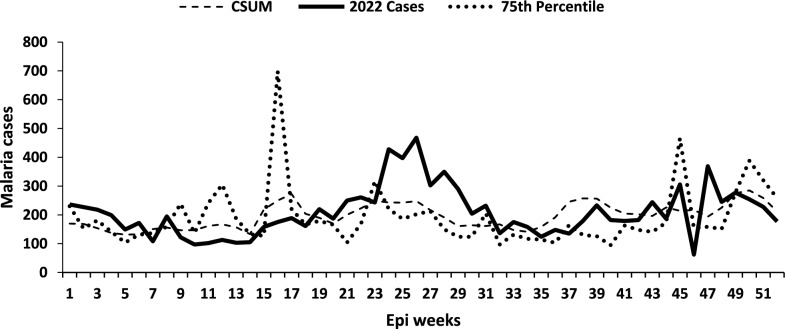
Weekly malaria cases on the currently used 75th percentile and C-SUM for the year 2022 for the low transmission Kisoro District in Southwestern Region, Uganda. This image shows clearly how the C-SUM method smooths out outliers in the data

For Kisoro District, Kigezi Region, an area of very low transmission also targeted for malaria elimination in the 2020–2025 Malaria Strategic Plan, the 75th percentile method detected 34 weeks above the threshold while the recommended C-SUM detected 26 weeks. This would have resulted in the district having a malaria outbreak requiring epidemic investigation from weeks 3–6 and 21–33 in 2022 using the C-SUM method, and weeks 3–6, 21–22, and 26–43 using the 75th percentile method (Fig. 5).

## Discussion

Identifying the appropriate situations to respond to an apparent increase in cases of a disease in an endemic setting is challenging. The use of transmission intensity-specific thresholds, based on historical data, is meant to facilitate the identification of malaria outbreaks and distinguish true increases from seasonal upsurges in endemic areas. Using real examples from Uganda, major differences between threshold calculation approaches in terms of the number of weeks above the threshold detected as well as the number of outbreaks that would require epidemic response were identified. Specifically, two approaches that are both meant to be acceptable for outbreak detection in medium-to-high transmission areas (mean + 2SD and 75th percentile) yielded large differences in the number of outbreak weeks detected across all levels of transmission. The 75th percentile method yielded outbreak weeks more similar to those identified by the very sensitive C-SUM method across all transmission levels. In addition, the true transmission levels in districts were often not reflective of the region to which they were assigned.

Both the 75th percentile and mean + 2SD methods have been recommended for malaria outbreak detection in medium- to high-transmission areas, suggesting their comparability and possible interchangeability. However, significant differences in the number of weeks exceeding the outbreak threshold between these two methods were identified, with the mean + 2SD method identifying significantly fewer outbreak weeks. A Kenyan study in three different regions similarly found that the 75th percentile method identified approximately 3 times as many months as being ‘epidemic’ as the mean + 2SD method [12]. Clear guidance on the application of these methods for specific transmission areas is required for improved malaria outbreak surveillance and detection.

While only the C-SUM method is recommended for low- or very low-transmission areas, no significant difference in the number of weeks above the threshold detected by the 75th percentile and C-SUM methods in these districts was observed. Existing guidance discourages the use of the 75th percentile method in low- and very low-transmission areas due to the potential for missing outbreaks [4, 13, 14]. In this evaluation, outbreaks were not missed. However, in medium- and high-transmission areas, the C-SUM method detected significantly more outbreak weeks than the 75th percentile method. This supports not using the C-SUM method in medium- and high-transmission areas to avoid false alarms, as it does not account for seasonal peaks [4]. Studies conducted in Sudan and Ethiopia for early malaria epidemic detection have suggested the use of both the 75th percentile and C-SUM methods as pre-malaria-outbreak warnings in areas with medium to high malaria transmission [15, 16].

The comparable sensitivity of the 75th percentile method and the C-SUM method in very low- and low-transmission areas and the significant differences observed in medium to high transmission areas suggests that the 75th percentile method could be applicable across all transmission levels. Since one objective of surveillance is the timely detection of outbreaks, the sensitivity of the 75th percentile method would provide timely detection of malaria epidemics, especially in medium- and high-malaria transmission areas. However, the use of this approach yielded more outbreaks than were feasible to respond to in Uganda during 2022. Thus, it may be useful to consider whether an alternate, less sensitive approach, such as the mean + SD method, could be applied for epidemic response prioritization when the 75th percentile yields more outbreak districts than can be adequately addressed with existing resources.

On adjustment of the 75th percentile to the 85th percentile, no statistically significant difference was observed in the number of outbreak weeks for low and medium transmission areas. Other studies have proposed adjusting the 75th percentile to the 90th percentile instead of the 85th to better accommodate malaria seasonal peaks and improve outbreak detection [4, 17–19]. However, the small differences in outbreak weeks detected between the 75th percentile and the 85Th percentile might not suffice to recommend this adjustment for better accommodation of seasonal peaks. It may be useful to consider other modified approaches, such as modifying the 75th percentile to the 90th percentile to better accommodate seasonal peaks in some situations.

On adjustment of the C-SUM method to the C-SUM + 2SD method, there was a significant decrease in the number of outbreak weeks detected, but no difference from the number of outbreak weeks detected by the mean + 2SD method. This similarity can be attributed to both methods using averages, with the main difference lying in their respective methodologies (the mean + 2SD method takes the mean number of cases for that week over the last five years and adds 2SD to establish the threshold. The C-SUM + 2SD method takes the running average of cases for the current epi week, the previous week, and the week after over the past 5 years and adds 2SD to establish a threshold). Similar findings were observed in Madagascar in a study analysing trends and forecasting malaria epidemics using a sentinel surveillance network which indicated improved specificity when the 2SD is added to the C-SUM [17]. A consideration of C-SUM + 2SD for epidemic detection in medium to high malaria transmission districts could provide an alternative method for malaria epidemic detection to the mean + 2SD method.

In Uganda, transmission levels, on which threshold approaches are meant to be based, are assessed using regional (larger; n = 15 in Uganda) data rather than the district (smaller; n = 146 in Uganda) data. Granularity in the actual malaria transmission levels, different from the regional transmission levels for the districts evaluated was identified. The study revealed notable differences in the malaria transmission of the evaluated districts and their nationally allocated regional malaria transmission levels. Districts in high-transmission regions were found to have medium- or low-transmission levels, while some districts in low-or very low-transmission regions had medium-transmission levels. These findings highlight the need for stratification of the malaria burden at district level rather than regional level. Stratification at district level could be helpful for instances when prioritization for epidemic response is required as it only applies to medium and high transmission areas. This could also support appropriate allocation of resources for improved malaria epidemic surveillance and response at district level.

### Limitations

The study's limitations include the absence of a definitive gold standard approach for identifying outbreaks; however, this is inherent to a highly endemic setting for any disease. Additionally, methods were evaluated in only 16 out of 146 districts in Uganda due to under-reporting by most districts. However, the selected districts were distributed around the country and across all transmission levels, which may enhance the generalizability of the study findings.

## Conclusion

Our study demonstrated notable differences in district malaria transmission levels from the assigned regional malaria transmission levels. Among the districts evaluated, the 75th percentile approach proved most applicable for all transmission areas. However, the number of epidemic weeks detected for medium- and high-transmission areas was significantly higher than the mean + 2SD method. This would challenge response in resource-limited settings which is the majority of Africa where the malaria burden is high. We recommend use of the 75th percentile method for epidemic detection in all malaria transmission areas and the use of mean + 2SD for prioritization of districts for response in situations of low resources. Furthermore, the stratification of areas to the smallest geographical unit possible would ensure detection of localized malaria outbreaks. Additionally, re-calculation of malaria transmission levels at district level and re-categorization of districts rather than regions would ensure appropriate malaria outbreak surveillance and detection for appropriate response.

## Data Availability

The datasets upon which our findings are based belong to the Uganda Ministry of Health. However, the datasets can be availed upon reasonable request from the corresponding author and with permission from the Uganda Public Health Fellowship Program.

## Abbreviations

API: Annual parasite incidence
DHIS2: District Health Information System (DHIS2)
HC: Health facility
SD: Standard deviation
C-SUM: Cumulative sum
HMIS: Health Management Information System
